# Progress on aspiration assessment methods for patients after esophageal cancer surgery in early: A review

**DOI:** 10.1097/MD.0000000000041214

**Published:** 2025-01-17

**Authors:** Yushuang Su, Yan Li, Zhongbin Chen, Hong Gao, Yaxie He, Xiaohua Li, Xiaying Zeng, Wei Lan, Qin Yang

**Affiliations:** a Department of Thoracic Surgery, Sichuan Provincial People’s Hospital, School of Medicine, University of Electronic Science and Technology of China, Chengdu, China; b Department of Nephrology, Sichuan Provincial People’s Hospital, School of Medicine, University of Electronic Science and Technology of China, Chengdu, China; c Department of ICU, Sichuan Provincial People’s Hospital, School of Medicine, University of Electronic Science and Technology of China, Chengdu, China; d School of Medicine, University of Electronic Science and Technology of China, Chengdu, China.

**Keywords:** assessment, dysphagia, esophageal cancer, esophagectomy, swallowing evaluation

## Abstract

Esophageal cancer is a relatively common malignant tumor of the digestive tract. Patients with esophageal cancer show a high incidence of aspiration after surgery, which has a serious impact on their prognosis and rehabilitation. Nevertheless, while existing and past endeavors have concentrated on enhancing the diagnostic and therapeutic strategies for esophageal cancer, the necessity of preventing pneumonia caused by postoperative aspiration remains to be adequately addressed. We compiled the presently published literature and offer the latest developments on the causes of postoperative aspiration in patients with esophageal cancer, screening methods, and swallowing assessment tools. Relevant published papers were collected through a search of the China national knowledge infrastructure, Ovid EMBASE, Web of Science, Cochrane, and PubMed databases. There are various methods for assessing swallowing function after surgery for esophageal cancer. Clinically, it is necessary to select appropriate assessment tools for the swallowing function. Research indicates that the application of risk prediction models can better assess aspiration in patients after esophageal cancer surgery, bridge gaps in qualitative analysis, and alter the clinical outcomes of patients. Predictive models for dysphagia screening in patients after esophagectomy have significant clinical advantages and exhibit good clinical applicability.

## 1. Introduction

Esophageal cancer (EN) is a common cancer of the digestive tract that, arises in the esophagus or esophageal junction,^[1]^ with the seventh highest incidence and sixth highest mortality rates of cancers worldwide,^[2]^ posing a serious threat to human health. The initial manifestation of esophageal cancer is difficult to detect. Therefore, most patients are already at a progressive stage of the disease at the time of diagnosis and treatment.^[3]^ Surgical treatment is the preferred treatment for patients with esophageal cancer. During surgery, a portion of the gastric tissue must be removed to create a gastric tube, which is then anastomosed to the preserved esophagus.^[4]^ Consequently, the patient must fast immediately after surgery until anastomotic leakage is ruled out. During this period, along with intravenous fluid replacement, intestinal nutritional supplements should be administered via enteral feeding tubes. Owing to the removal of a portion of the gastroesophagus and reestablishment of the upper gastrointestinal tract, the vagus nerve is damaged after gastric substitution esophageal surgery, which affects the normal physiology and functional integrity of the esophagus. After surgery, all patients experienced different degrees of decline in gastroesophageal reflux, peristalsis of the residual esophagus, and esophageal contouring ability^[5]^ and some patients had weakened hyoid bone movement, epiglottis rotation, and prolonged pharyngeal response.^[6]^ This suggests that the patient’s swallowing function is affected to varying degrees. The latest guidelines issued by the American Heart Association and the Chinese Dysphagia Dietary and Nutritional Management Expert Consensus Group^[7,8]^ indicate that an assessment of the risk of aspiration should be performed before the patient is ready to eat or drink.

Dysphagia is 1 of the main factors that trigger aspiration. The aspiration is a symptom that is defined as the inhalation of oropharyngeal or gastric contents into the lower respiratory tract,^[9]^ judging by the clinical manifestations after aspiration, it is divided into visible aspiration and invisible aspiration.^[10]^ After aspiration, epithelial cells and alveolar macrophages produce chemical signaling agents that activate neutrophils to release proteases and reactive oxygen species, which ultimately damage alveolar capillary units.^[11]^ We surmised that aspiration is a potentially fatal complication of hidden nature. Studies have shown that the incidence of aspiration is higher in the early postoperative period than in the later stages of the disease; 44% of patients with aspiration may develop pneumonia,^[12]^ and deaths due to pneumonia and respiratory failure account for 33.1% of patients who die after esophageal surgery.^[13]^ As the severity of dysphagia gradually increases, its impact on the patients becomes more significant. Compared to non-aspiration patients, hospitalization costs for patients with aspiration increased by $49,372, and the length of stay was 43% longer.^[14]^ Studies have found that the incidence of early postoperative aspiration in patients with esophageal cancer is 32% to 48%.^[6,15,16]^ Clarifying the patient’s postoperative swallowing function status and its influencing factors is crucial for postoperative safety and survival. However, significant heterogeneity was observed in the factors influencing aspiration and assessment methods for patients. This study analyzed the relevant literature on swallowing assessment tools for patients following esophageal cancer resection, discussed the causes of aspiration, relevant assessment tools, and factors, and provided a reference for swallowing assessment in patients after esophageal cancer resection.

## 2. Methods

This narrative review synthesized clinical trials, fundamental research studies, and overviews focusing on methods for assessing aspiration after esophagectomy.

### 2.1. Research methodology

The included guidelines and literature satisfied the following criteria. The inclusion criteria: the study subjects patients with esophageal cancer, aged ≥ 18 years; treatment method: radical esophagectomy; assessment of influencing factors or swallowing function; study designs including randomized controlled trials, quasi-experimental studies, cross-sectional surveys, cohort studies, case-control studies, and mixed-methods studies. The exclusion criteria: dysphagia caused by tumor recurrence or anastomotic stricture after surgery; had previous tracheotomy.

A comprehensive literature search was conducted across multiple databases, including the China national knowledge infrastructure, Ovid EMBASE, Web of Science, Cochrane, and PubMed, from the inception of each database to September 2024. The medical subject headings listed below were integrated: “esophageal cancer or esophageal neoplasms or esophagus or cancer of esophagus, esophagus cancer,” “esophagectom* or oesophagectom*,” and “dysphagia or aspiration or swallowing disorder*.” Limit Chinese papers to those published in core journals.

### 2.2. Process of study selection

We conducted a search for pertinent articles and subsequently screened them for further reading based on their titles and abstracts. All studies included in this review were thoroughly discussed. Ethical approval was not required for this narrative review because it did not use human or patient data.

### 2.3. Data extraction

Information was extracted from the included literature, including the authors, study design, sample size, swallowing function assessment, screening methods, and influencing factors.

## 3. Result

### 3.1. Study selection and characteristics

A total of 32 articles were ultimately included in this study.^[6,12,15–44]^ Characteristics of included studies is shown in Table 1, including the authors, study design, and swallowing function assessment. Table 2 shows a detailed description of the screening methods and influencing factors.

**Table 1:** Characteristics of included studies.

Author	Country	Year	Study design	Total patient	Dysphagia/aspiration (%)	Swallowing assessment time	Assessment tool
Kim^[6]^	Korea	2015	Case–control	47	NA/48.9	Postoperative	VFSS, PAS
Berry^[12]^	USA	2010	Retrospective survey	379	NA/12.0	Before initiation of oral feedings	VFSS
Lee^[15]^	Korea	2016	Retrospective survey	118	NA/32.2	Postoperative days 7 to 10	VFSS
Kumai^[16]^	Japan	2016	Retrospective survey	25	44/36	Postoperative weeks 2 to 3	VFSS, PAS, FEES
Blakely^[17]^	USA	2022	Retrospective survey	50	NA/22	Postoperative 2-wk	VFSS
Kurita^[18]^	Japan	2022	Retrospective survey	201	NA/18.9	Postoperative day 6 or 7	VFSS, PAS
Mann^[19]^	USA	2022	Retrospective survey	129	35.6/NA	Before initiation of oral feedings	VFSS, PAS
Kojima^[20]^	Japan	2022	Retrospective survey	216	NA/13.4	Postoperative day 6 or 7	VFSS, PAS
Mafune^[21]^	Japan	2019	Retrospective survey	32	34.4/15.6	Postoperative day 7	VFSS
Kawata^[22]^	Japan	2024	Retrospective survey	114	34.0/20.0	Postoperative 7 to 10 d	VFSS, PAS
Yasuda (2014)^[23]^	Japan	2014	Retrospective survey	30	NA/20.0	Postoperative day 7	FEES
Katsumata^[24]^	Japan	2019	Retrospective survey	54	22.2/NA	Postoperative day 7	VFSS
Hiwatari^[25]^	Japan	2020	Retrospective survey	72	NA/23.6	Postoperative day 7	VFSS
Yasuda (2013)^[26]^	Japan	2013	Prospective survey	40	35/20	Before initiation of oral feedings	VFSS
Takatsu (2024)^[27]^	Japan	2024	Retrospective survey	312	41.6/NA	Postoperative day 8 or later	VFSS, PAS
Mayanagi^[28]^	Japan	2021	Retrospective survey	187	NA	Postoperative days 7 to 15	VFSS, FEES, FILS
Sugase^[29]^	Japan	2022	Retrospective survey	304	36.8/35.5	Postoperative days 8 to 9	VFSS
Yokoi^[30]^	Japan	2019	Prospective survey	59	NA/10.2	Postoperative days 14	RSST
Takatsu (2020)^[31]^	Japan	2020	Quasi-experimental	276	NA/21.7	Postoperative days 5 or 6	MWST
Yang^[32]^	China	2020	RCT	237	NA/19.4	Postoperative day 7	WST
Lewin^[33]^	USA	2001	Self before-and-after comparison	26	NA/81	Postoperative day 13	VFSS, PAS
Koh^[34]^	Canada	2004	Prospective survey	9	22.2/0.0	Postoperative 6 to 40 mo	VFSS, PAS
Okumura^[35]^	Japan	2015	Quasi-experimental	26	NA	Postoperative days 7 to 10	VFSS, PAS
Yuen^[36]^	China	2018	Prospective survey	29	73/28	Postoperative more than 6 mo	VFSS, PAS, VDS
Easterling^[37]^	USA	2000	Prospective survey	8	62.5/37.5	Postoperative days 7 to 10	VFSS, PAS
Pierie^[38]^	Netherlands	2000	Retrospective survey	22	32/NA	Postoperative weeks 1 to 16	Functional Dysphagia Score
Kato^[3^^9^^]^	Japan	2007	Retrospective survey	27	NA	Postoperative days 12 to 21	VFSS
Wang^[40]^	China	2021	Quasi-experimental	100	NA	Before discharge	SSA
Yue^[41]^	China	2018	RCT	60	NA	Postoperative weeks 4	SSA
Hou^[42]^	China	2017	RCT	46	NA	NA	WST
Liu^[43]^	China	2019	Mixed methods	NA	NA	NA	Self-developed
Oguma^[44]^	Japan	2022	Systematic reviews	942	NA	NA	VFSS, PAS

Abbreviations: FEES = fiberoptic endoscopic evaluation of swallowing, FILS = food intake level scale, MWST = modified water swallow test, NA = not available, RSST = repetitive saliva swallowing test, SSA = standardized swallowing assessment, WST = water swallow test.

**Table 2:** Screening methods and influencing factors.

Author	Method for factors	Significant factors
Kim^[6]^	Univariate analysis, Pearson correlation test	Prolonged operation time, decreased movement of the hyoid and rotation of the epiglottis
Berry^[12]^	Univariate analysis, multivariate logistic analysis	Age, cervical anastomosis
Lee^[15]^	Univariate analysis, multivariate logistic analysis	Operative time, RLNP
Kumai^[16]^	Pearson correlation test, univariate analysis, multivariate logistic analysis	Laryngeal elevation
Blakely^[17]^	Univariate analysis, multivariate logistic analysis	Age
Kurita^[18]^	Univariate analysis, multivariate logistic analysis	Recurrent laryngeal nerve palsy, location, handgrip strength, age
Mann^[19]^	Univariate analysis, multivariate logistic analysis	Age, preoperative feeding tube
Kojima^[20]^	Univariate analysis, multivariate logistic analysis	PRLNP, Tongue pressure
Mafune^[21]^	Univariate analysis, multivariate logistic analysis	Cervical lymph node dissection
Kawata^[22]^	Univariate analysis, multivariate logistic analysis	RLNP, GHMA
Yasuda (2014)^[23]^	Univariate analysis, multivariate logistic analysis	Plasma substance P
Katsumata^[24]^	Univariate analysis	Cervical lymph node dissection, CSA-PMM, CSA-GH
Hiwatari^[25]^	Univariate analysis	CSA-GH change rate, LED difference, LED change rate
Yasuda (2013)^[26]^	Univariate analysis, multivariate logistic analysis	Age, laryngeal elevation
Takatsu (2024)^[27]^	Univariate analysis, multivariate logistic analysis	PNI, BMI, maximum diagonal hyoid bone elevation
Mayanagi^[28]^	Univariate analysis, multivariate logistic analysis	RLNP, SMI
Sugase^[29]^	Univariate analysis, multivariate logistic analysis	Age, BMI, RLNP
Yokoi^[30]^	Pearson correlation test, univariate analysis, multivariate logistic analysis	Change in tongue pressure
Easterling^[37]^	Univariate analysis	Anterior hyoid, superior hyoid
Yuen^[36]^	Univariate analysis, Pearson correlation test	Pathological N, age, males
Koh^[34]^	Univariate analysis	Esophageal pressures
Pierie^[38]^	Univariate analysis	Vocal cord palsy
Kato^[39]^	Univariate analysis	Retrosternal route
Liu^[43]^	Delphi	Personal factors, surgical factors, enteral Nutrition management, oral feeding, special examinations.
Oguma^[44]^	Systematic reviews	Preoperative sarcopenia

Abbreviations: BMI = body mass index, CSA-PMM = cross-sectional area of the psoas major muscle, GHMA = geniohyoid muscle midsagittal area, LED = laryngeal elevation distance, MADH = maximal anterior displacement of the hyoid, PNI = prognostic nutritional index, RLNP = recurrent laryngeal nerve palsy, SMI = skeletal muscle index, VC = vital capacity.

### 3.2. Assessment of swallowing function in patients after esophageal cancer surgery

#### 3.2.1. Swallowing test

The swallowing test assesses the patient’s swallowing function by observing whether symptoms such as choking or aspiration, occur when swallowing solids or liquids. According to the relevant literature, oral intake tests commonly used in foreign countries to assess the risk of aspiration include the Saliva swallowing test (RSST) and water swallow test (WST),^[32,42]^ which is 1 of the most classic screening methods for assessing swallowing function. This test evaluates the presence of swallowing disorders by observing the number of swallows required by the subject to swallow 30 mL of water and whether choking occurred during the process. Based on the test results, swallowing ability can be categorized into 5 grades. More than 3 grades indicated that the patient had dysphagia, but the test had poor sensitivity to silent aspiration.^[45]^ Studies have shown that patients may experience varying degrees of dysphagia after esophageal cancer surgery.^[5]^ Therefore, some studies^[31]^ have adopted a modified WST to assess swallowing function in these patients, which involves reducing the liquid volume to 3 ml. The standardized swallowing assessment (SSA) scale enables the integration of clinical examination and WST, and is used to assess aspiration in patients with impaired swallowing.^[46]^ In China, some experimental studies^[40,41]^ have used SSA to evaluate the swallowing function of patients after esophagectomy. The scores were primarily based on the patient’s swallowing condition, such as the presence or absence of choking. Higher scores indicated poorer swallowing function. A positive result was indicated by choking while drinking water, suggesting the presence of swallowing disorder. Its advantages include ease of use and low costs. This method does not require special equipment or the involvement of speech therapists. Healthcare professionals can implement this after receiving training. One study^[38]^ used the functional dysphagia scale, focusing on whether patients reported symptoms of swallowing difficulty during meals. This assessment method categorizes patients based on their eating habits and emphasizes their subjective experience of swallowing. The repetitive saliva swallowing test (RSST) was conducted by an oral surgeon who counted the number of times a patient swallowed saliva within 30s, with a cutoff value of 3 swallows. Previous studies have shown that this test has low specificity for predicting dysphagia. Yokoi^[30]^ used this method for swallowing assessment. This test does not require the ingestion of any food, thus eliminating concerns about the need for fasting due to anastomotic leakage, and can be performed early after surgery. Liu^[43]^ developed an aspiration assessment scale for patients with esophageal cancer using the Delphi method. However, this scale has not yet been validated.

#### 3.2.2. Instrumental diagnosis

Because the clinical manifestations of silent aspiration are not obvious, auxiliary equipment is required for the diagnosis. A video fluoroscopic swallowing study (VFSS) is considered the gold standard for swallowing function tests.^[47]^ This examination requires patients to swallow barium-containing foods with different textures and volumes to assess their swallowing function comprehensively. However, there is a relatively high risk of aspiration during the examination process. In addition, VFSS is also used in conjunction with the penetration-aspiration scale (PAS).^[6,16,18–20,22,27,33–37]^ The total PAS score ranged from 0 to 8, with higher scores indicating a higher risk of aspiration. Fiberoptic endoscopic examination of swallowing (FEES) can identify the location of food residues during eating, detect aspiration, and directly observe recurrent laryngeal nerve palsy (RLNP). It is also used in patients with esophageal cancer.^[23]^ Digital X-ray imaging of the pharynx, esophagus, and gastric cardia with a contrast agent: The patient was instructed to drink the contrast agent, and simultaneously use X-rays to dynamically observe the flow of the contrast agent during the swallowing process. Although not as precise as the video recording equipment, its rapid capture capability can generally serve as a substitute for motion image recording to observe pharyngeal movements. This examination method can also provide information on aspiration during the swallowing process.^[48–50]^ However, the high cost and complexity of the procedure make it difficult for both the patient and the hospital to conduct the examination.

### 3.3. Screening method for aspiration risk factors for patients after esophagectomy

#### 3.3.1. Logistic regression method

Logistic regression is a generalized linear model that predicts the probability of future events by analyzing existing data to select disease risk factors.^[51]^ Mayanagi et al^[28]^ concluded that preoperative hyposmia and RLNP were independent risk factors for postoperative dysphagia after esophageal cancer using logistic regression analysis in 187 postoperative patients, and Lee et al^[15]^ analyzed the factors related to postoperative dysphagia in 118 patients using logistic regression analysis, and found that prolonged surgical time and RLNP were independent risk factors for postoperative dysphagia. Berry^[12]^ used the same method to conclude that age and anastomotic site were independent risk factors. In addition, Kato^[39]^ found that digestive tract reconstruction is related to postoperative dysphagia, possibly due to impaired hyoid bone movement and laryngeal elevation. Posterior mediastinal reconstruction should be performed whenever possible. The factors screened by the logistic regression analysis were derived from empirical research, which is highly scientific.

#### 3.3.2. Systematic review method

The systematic review method involves screening a series of studies using rigorous evaluation of the literature, the results of which are qualitative or quantitative methods to derive reliability.^[52]^ A systematic review published by Steele et al^[53]^ showed that an increased baseline respiratory rate, decreased oxygen saturation, decreased hyoid elevation, and decreased lung capacity are physiological factors related to the influence of malabsorption. A systematic review published by Chen et al^[54]^ stated that intraoperative recurrent laryngeal nerve monitoring reduces the incidence of malabsorption in postoperative patients with esophageal cancer. Oguma et al^[44]^ used a systematic evaluation approach to conclude that preoperative skeletal muscle index was associated with postoperative dysphagia. These methods can maximize the application of published or unpublished studies to screen impact factors and increase the amount of available research data.

#### 3.3.3. Delphi method

The Delphi method, 1 of the ways to assess the research object by formulating a questionnaire, oriented to a team of experts to obtain opinions as a guideline, converging and integrating multiple viewpoints, and synthesizing experts’ opinions, is regarded as a representative of qualitative assessment.^[55]^ Liu ^[43]^ constructed a postoperative esophageal cancer aspiration assessment scale based on the Delphi method, including 5 primary indicators and 30 secondary indicators, and the patient’s own factors, surgical anesthesia factors, indwelling tubes, hoarseness, and examination factors were the risk factors for postoperative aspiration in patients with esophageal cancer. The Delphi method is convenient and clear, and fully exploits the power of synergistic body wisdom to realize the prediction without the derivation of data equations.

## 4. Discussion and prospect

The differences are significant in the results of studies related to these people, mainly because of the heterogeneity of geographic regions in related studies, assessment tools for aspiration, and the level of influencing factors covered in the studies. Geographical differences: esophageal squamous cell carcinoma accounts for 90% of esophageal cancer incidence in China, whereas adenocarcinoma is predominant in North America, Europe, and the United States. Dysphagia and weight loss are more prevalent in patients with squamous carcinoma, accounting for approximately 93% of all total.^[2]^ There are significant differences in the cause of pathogenesis, site of tumor growth, treatment methods, and survival cycle; different assessment tools for aspiration: the diagnostic criteria for aspiration adopted in the current study including WST, FEES, VFSS, and SSA; and wide differences in factors: different screening methods for risk factors have their own advantages and disadvantages in selecting risk factors for aspiration.

Evaluation of dysphagia may combine multiple approaches. Assessment methods such as the WST, RSST, and SSA, compared to the VFSS, are more convenient to operate and do not require specialized equipment or preparations. However, they exhibit lower sensitivity. The risk factors for aspiration in patients with esophageal cancer included: self-factors, nutritional factors, surgical factors, postoperative signs, pipeline factors, and other factors. Numerous influencing factors affect postoperative aspiration, and comprehensive multidisciplinary judgment must be integrated. A risk prediction model is a mathematical equation that integrates a patient’s risk factors, based on data analysis and interpretation, to calculate the probability of a patient experiencing a specific event or outcome, thereby identifying high-risk individuals.^[56]^ Through continuous practice by researchers, risk prediction models have been proven to be accurate, convenient, and rapid assessment tools, and have been widely accepted by healthcare professionals. Bas et al^[57]^ used the functional oral intake scale (FOIS) and a self-assessment questionnaire to establish a prediction model for aspiration patients with dysphagia, with an ACU value of 0.92. Song et al^[58]^ used voice analysis to predict aspiration, and predicted better performance. Lee et al^[15]^ developed a predictive model for postoperative esophageal cancer with, a sensitivity of 68.4%. The study was conducted at a single center with a relatively small sample size. In future, a multicenter study should be conducted to increase the sample size and develop a better aspiration prediction model with improved prevention and control capabilities. Risk prediction for esophageal cancer patients before resumption of oral feeding after surgery is helpful for clinical nursing staff to identify patients at high risk of aspiration at an early stage, assist in the discovery of hidden risks, and enhance the safety of oral intake.

Dysphagia is a common symptom of esophageal cancer surgery. It is necessary to select a suitable swallowing function screening method for patients with esophageal cancer and establish a risk prediction model for postoperative dysphagia, so that high-risk patients can be identified and treated with swallowing function measures as early as possible. The Chinese Expert Consensus on Dietary and Nutritional Management of Dysphagia^[8]^ states that risk assessment should be performed before transoral feeding for people at high risk of dysphagia. Considering the promotion of economic and clinical applications, a combination of methods can be used for aspiration assessment. A prediction model can be constructed using logistic regression, random forest, decision tree, and other methods, based on a combination of scales and clinical indicators. Achieving precise individualized assessments is of great significance for the selection of diagnostic and intervention methods. It can bridge defects in qualitative analysis and improve the early warning of the risk of postoperative aspiration in patients with esophageal cancer. Based on controllable key factors, swallowing function training is covered in the care of postoperative patients at a high risk of aspiration, which will provide theoretical arguments and references for medical personnel to prevent the occurrence of postoperative oral feeding aspiration in patients with esophageal cancer. In the future, preventive measures and rehabilitation intervention programs should be developed based on predictive modeling, which is of great significance for effectively reducing the incidence of aspiration and pneumonia and promoting patient recovery. This study had several limitations. First, the methodological quality of the included studies was not assessed. Second, there may be potential bias in the literature selection process.

## Author contributions

**Conceptualization:** Yushuang Su, Xiaying Zeng.

**Investigation:** Yaxie He, Xiaying Zeng.

**Methodology:** Yan Li, Xiaying Zeng.

**Resources:** Yan Li.

**Software:** XiaoHua Li.

**Supervision:** Hong Gao, Yaxie He.

**Writing – original draft:** Yushuang Su, Yan Li.

**Writing – review & editing:** Yushuang Su, Zhongbin Chen, Wei Lan, Qin Yang.
